# Dual melanocortin-4 receptor and GLP-1 receptor agonism amplifies metabolic benefits in diet-induced obese mice

**DOI:** 10.15252/emmm.201404508

**Published:** 2015-02-04

**Authors:** Christoffer Clemmensen, Brian Finan, Katrin Fischer, Robby Zachariah Tom, Beata Legutko, Laura Sehrer, Daniela Heine, Niklas Grassl, Carola W Meyer, Bart Henderson, Susanna M Hofmann, Matthias H Tschöp, Lex HT Van der Ploeg, Timo D Müller

**Affiliations:** 1 Institute for Diabetes and Obesity & Helmholtz Diabetes Center, Helmholtz Zentrum München, German Research Center for Environmental Health (GmbH)Neuherberg, Germany; 2 Division of Metabolic Diseases, Department of Medicine, Technische Universität MünchenMunich, Germany; 3 Institute for Diabetes and Regeneration Research & Helmholtz Diabetes Center, Helmholtz Zentrum München, German Research Center for Environmental Health (GmbH)Neuherberg, Germany; 4 RhythmBoston, Massachusetts, USA

**Keywords:** diabetes, Glp-1r, liraglutide, Mc4r, obesity

## Abstract

We assessed the efficacy of simultaneous agonism at the glucagon-like peptide-1 receptor (GLP-1R) and the melanocortin-4 receptor (MC4R) for the treatment of obesity and diabetes in rodents. Diet-induced obese (DIO) mice were chronically treated with either the long-acting GLP-1R agonist liraglutide, the MC4R agonist RM-493 or a combination of RM-493 and liraglutide. Co-treatment of DIO mice with RM-493 and liraglutide improves body weight loss and enhances glycemic control and cholesterol metabolism beyond what can be achieved with either mono-therapy. The superior metabolic efficacy of this combination therapy is attributed to the anorectic and glycemic actions of both drugs, along with the ability of RM-493 to increase energy expenditure. Interestingly, compared to mice treated with liraglutide alone, hypothalamic *Glp-1r* expression was higher in mice treated with the combination therapy after both acute and chronic treatment. Further, RM-493 enhanced hypothalamic *Mc4r* expression. Hence, co-dosing with MC4R and GLP-1R agonists increases expression of each receptor, indicative of minimized receptor desensitization. Together, these findings suggest potential opportunities for employing combination treatments that comprise parallel MC4R and GLP-1R agonism for the treatment of obesity and diabetes.

## Introduction

The worldwide prevalence of obesity and its associated metabolic complications is an increasing threat to public health (Wang *et al*, 2011). As a result, the development of safe and effective pharmacotherapies is a global priority. The etiology of metabolic disease is complex, with diverse pathophysiological mechanisms, emphasizing the challenges inherent in the development of medicinal options for the disorder (Kopelman, 2000; Scheen & Van Gaal, 2014). This is exemplified by the poor success rate in the development of anti-obesity drugs, as less than a handful of pharmacotherapies for obesity have progressed through regulatory approval (Rueda-Clausen *et al*, 2013). Recent clinical and pre-clinical advances indicate that simultaneously targeting more than one signaling pathway could lead to superior metabolic efficacy and fewer adverse events compared to traditional mono-therapies (Greenway & Bray, 2010; Rodgers *et al*, 2012; Sadry & Drucker, 2013). Simultaneous targeting of multiple metabolic pathways can be realized either via co-administration of distinct hormones (Muller *et al*, 2012; Clemmensen *et al*, 2014) or through employing unimolecular co-agonists that combine and integrate several hormone action profiles as well as different modes of pharmacological action (Day *et al*, 2009; Pocai *et al*, 2009; Finan *et al*, 2012, 2013, 2015).

The melanocortin-4 receptor (MC4R) plays a seminal role in regulating energy metabolism (Tao, 2010). Stimulation of pro-opiomelanocortin (POMC) expressing neurons in the hypothalamic arcuate nucleus results in the synthesis, cleavage and release of several bioactive peptides, including the endogenous MC4R-agonists α-MSH, β-MSH and γ-MSH (Castro & Morrison, 1997). Ligand-induced activation of MC4R results in inhibition of food intake and stimulation of energy expenditure, promoting a negative energy balance (McMinn *et al*, 2000; Balthasar *et al*, 2005). These observations and the finding that a plethora of MC4R loss-of-function variants predispose to human obesity have made the MC4R an attractive target for the development of novel, anti-obesity pharmacotherapies (Huszar *et al*, 1997; Yeo *et al*, 1998; Farooqi *et al*, 2003). Furthermore, MC4R-agonism enhances peripheral insulin sensitivity and improves glucose tolerance in rodents and non-human primates (Obici *et al*, 2001; Kievit *et al*, 2013), implying that MC4R-based drug therapies hold promise for treatment of type 2 diabetes and obesity. Unfortunately, adverse effects, including increased blood pressure and heart rate, have hampered the clinical progression of MC4R-based therapeutics thus far (Fani *et al*, 2014). Nevertheless, a new MC4R agonist, RM-493 (previously BIM-22493), was recently shown to decrease adiposity, increase energy expenditure and improve glucose metabolism with no detrimental cardiovascular effects in DIO rhesus macaques (Kievit *et al*, 2013), providing proof of concept for the use of MC4R-agonists to safely treat the metabolic syndrome. RM-493 is a small synthetic peptide with a unique activity profile distinct from other clinically tested MC4R agonists, including LY-2112688, and is currently in phase IIA clinical trials for the treatment of obesity and the metabolic syndrome.

Glucagon-like peptide-1 receptor (GLP-1R) agonists represent the best-in-class pharmacotherapies for treating type 2 diabetes (Drucker & Nauck, 2006). Emerging evidence supports that GLP-1R agonism can lower body weight in obese subjects (Astrup *et al*, 2009; Madsbad, 2014), corroborating rodent studies. In humans, however, the anorectic efficacy of GLP-1 mimetics is modest (Vilsboll *et al*, 2012). Interestingly, recent studies have shown that integrating GLP-1R agonism with glucagon receptor agonism can enhance the weight-lowering properties of both entities in rodents (Day *et al*, 2009; Pocai *et al*, 2009) and humans (Cegla *et al*, 2014). Balancing glucagon action with incretin-based agonism adds a thermogenic dimension that elicits weight loss without compromising the anti-diabetic and anorexigenic properties residing within GLP-1R agonism. The concept of using co-agonists or the co-administration of two or more hormones with complementary pharmacological properties can be expanded to include a multitude of novel combinatorial treatment regimes. It is anticipated that these may represent valuable personalized treatment opportunities for the metabolic syndrome.

The aim of the present study was to evaluate the efficacy of adjunctive administration of the novel MC4R agonist RM-493 (currently in a phase 2 clinical trial) and the anti-diabetic GLP-1R agonist liraglutide to improve glucose and energy metabolism in DIO mice. Based on the efficacies of MC4R and GLP-1R agonists to lower body weight and to improve glycemic control through distinct signaling mechanisms, we hypothesized that co-administration of these compounds may produce metabolic benefits superior to what can be accomplished with corresponding mono-therapies. In line with our hypothesis, we report that co-treatment with RM-493 and liraglutide amplifies body weight loss and cholesterol metabolism and enhances glycemic control in DIO mice. The augmented metabolic efficacy was attributed to unique actions of liraglutide and RM-493 to reduce caloric intake, increase energy expenditure and improve cholesterol and glucose control. This is the first study to demonstrate significantly enhanced efficacy that results from combining MC4R and GLP-1R agonism for the treatment of obesity and diabetes.

## Results

### RM-493 and liraglutide co-treatment enhances weight loss in DIO mice

**Figure 1:**
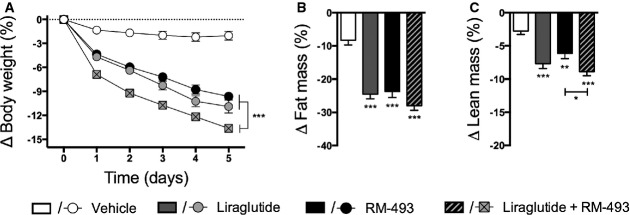
Effect of liraglutide and RM-493 co-treatment on body weight and body composition in DIO mice
A-C Five-day treatment of DIO male mice with vehicle (white), liraglutide (10 nmol/kg) (gray), RM-493 (3.6 μmol/kg) (black), or liraglutide (10 nmol/kg) and RM-493 (3.6 μmol/kg) (checkered). Effects on (A) body weight and (B, C) body composition. Compounds were administered by daily subcutaneous injections. Data represent means ± SEM;*n *=* *8; *P < 0.05, **P < 0.01, ***P < 0.001.

To test if simultaneous targeting of GLP-1R and MC4R promotes metabolic benefits beyond the respective mono-therapies, 28-week-old male DIO mice (51.92 ± 0.84 g) were treated for 5 consecutive days with liraglutide (10 nmol/kg/day), RM-493 (3.6 μmol/kg/day), the combination of liraglutide (10 nmol/kg/day) and RM-493 (3.6 μmol/kg/day) or vehicle control. Based on an *in vivo* dose titration with liraglutide and RM-493, dosages were selected to evaluate whether adjunctive therapy exceeds the benefits of each mono-therapy. Five days of treatment with either mono-therapy significantly decreased body weight relative to vehicle controls (*P* < 0.001) (Fig1A and Supplementary Fig S1A). As expected from dose selection, there was no difference in weight reduction between mice treated with liraglutide and mice treated with RM-493 alone (Fig1A and Supplementary Fig S1A). However, co-treatment with liraglutide and RM-493 resulted in a greater weight loss relative to mice treated with either mono-therapy (*P* < 0.001 relative to both mono-therapies) (Fig1A) which was reflected in minor body composition alterations (Fig1B and C and Supplementary Fig S1B and C).

### The metabolic benefits of RM-493 and liraglutide co-administration are linked to parallel changes in food intake and energy expenditure

**Figure 2:**
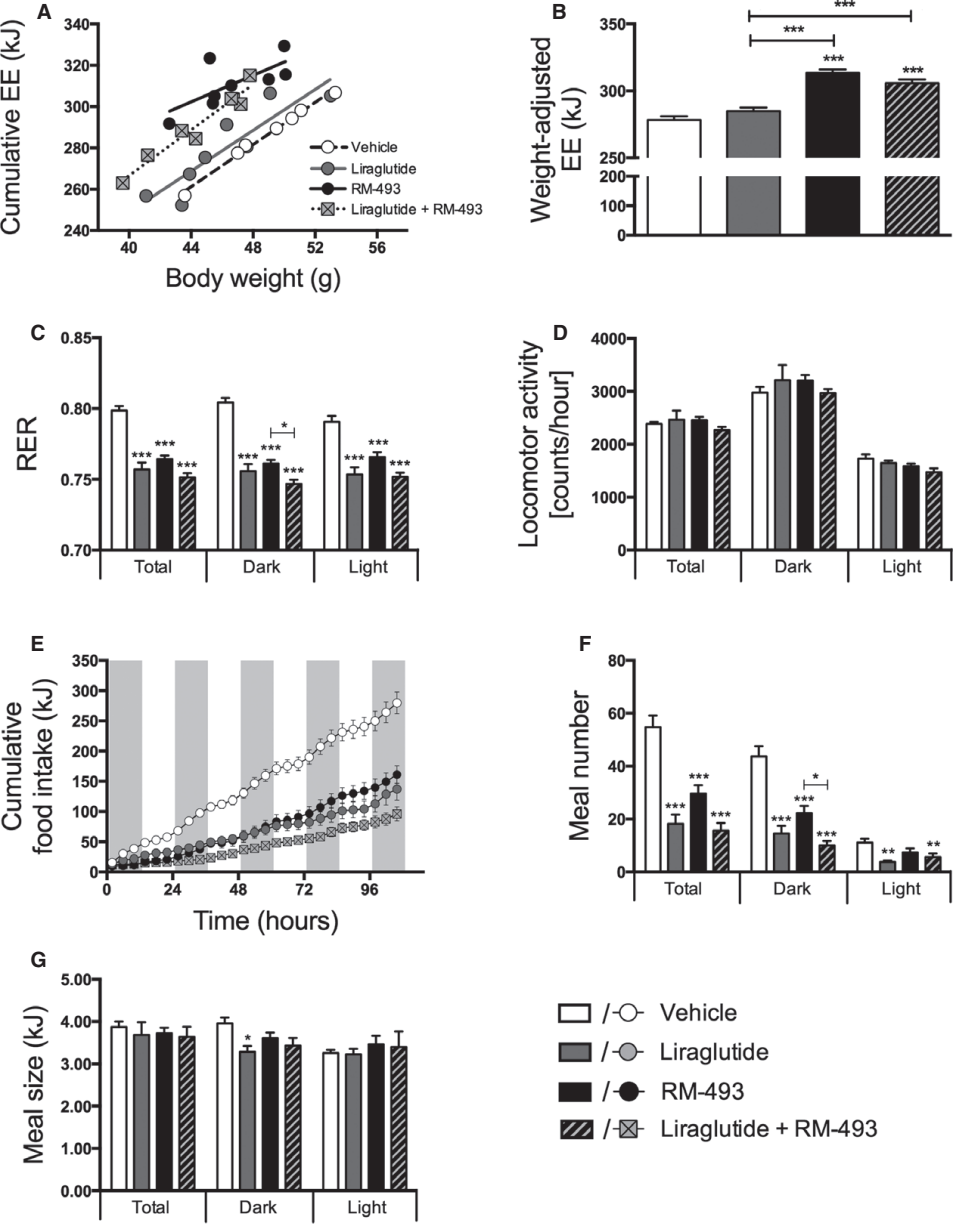
Effect of liraglutide and RM-493 co-treatment on energy metabolic parameters in DIO mice
A-G Five-day treatment of DIO male mice with vehicle (white), liraglutide (10 nmol/kg) (gray), RM-493 (3.6 μmol/kg) (black), or liraglutide (10 nmol/kg) and RM-493 (3.6 μmol/kg) (checkered). Effects on (A, B) energy expenditure, (C) respiratory exchange ratio (RER), (D) locomotor activity, (E) cumulative food intake, (F) meal number and (G) meal size. Compounds were administered by daily subcutaneous injections. Data represent means ± SEM;*n *=* *8; *P < 0.05, **P < 0.01, ***P < 0.001.

In line with previous reports showing that MC4R agonism promotes energy expenditure (Chen *et al*, 2000), we observed amplified energy expenditure in mice treated with RM-493 alone and in mice treated with the combination of RM-493 and liraglutide relative to both vehicle-treated mice and mice treated with liraglutide alone (*P* < 0.001) (Fig2A and B). Thus, the induction of energy expenditure is attributed to the presence of MC4R activity. In accordance with decreased body fat mass, both mono-therapies and the combination therapy yielded a similar reduction in the respiratory exchange ratio (RER) relative to vehicle-treated controls (*P* < 0.001), indicating a shift toward higher lipid utilization (Fig2C). Importantly, the RM-493-induced increase in energy expenditure was not reflected in changes in locomotor activity (Fig2D). Both RM-493 and liraglutide mono-therapy decreased caloric intake relative to vehicle controls (Fig2E). Interestingly, the combination therapy induced a greater reduction in food intake compared to both RM-493 (*P* < 0.01) and liraglutide (*P* < 0.05) mono-therapy. All compound treatments decreased the number of voluntary meals consumed (Fig2F) rather than the meal size (Fig2G). Thus, the co-administration of RM-493 and liraglutide not only profits from MC4R-mediated amplification of thermogenic pathways but it also benefits from the unique anorexigenic efficacy of each individual compound.

**Figure 3:**
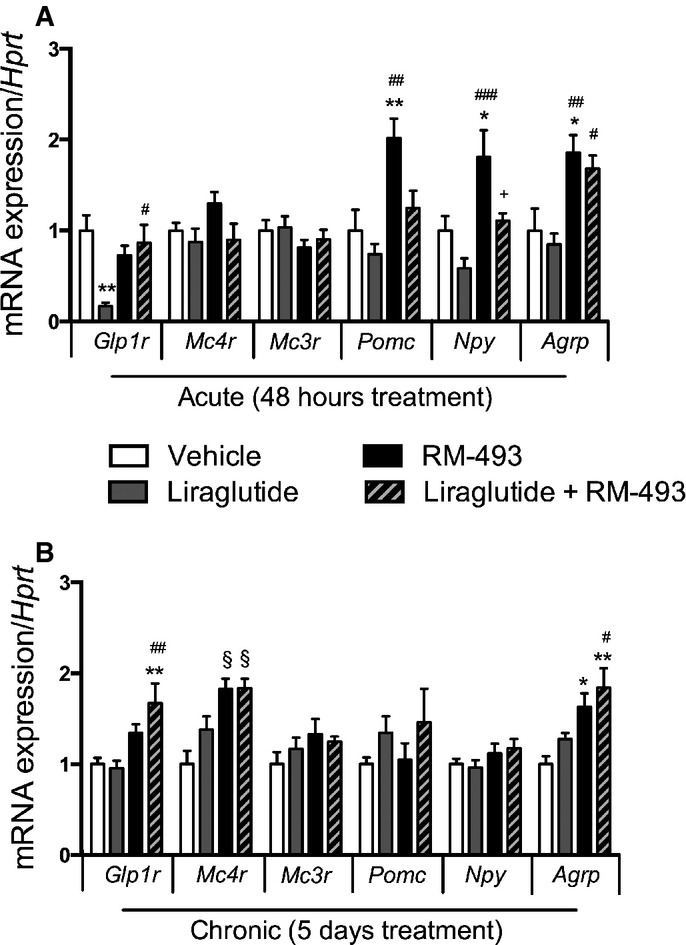
Effect of liraglutide and RM-493 co-treatment on hypothalamic gene expression in DIO mice
A, B Treatment-induced changes in hypothalamic gene expression in DIO mice treated for 2 (A) or 5 (B) days with vehicle (white), liraglutide (10 nmol/kg) (gray), RM-493 (3.6 μmol/kg) (black), or liraglutide (10 nmol/kg) and RM-493 (3.6 μmol/kg) (checkered). Compounds were administered by daily subcutaneous injections, and the last injection was provided 2 h prior to tissues sampling. Data represent means ± SEM;*n *=* *8; *P < 0.05, **P < 0.01, ^§^*P* < 0,001, comparison between treatment and vehicle control. ^#^*P* < 0.05, ^##^*P* < 0.01, ^###^*P* < 0.001, comparison to liraglutide. ^+^*P* < 0.05, comparison to RM-493.

We explored the molecular underpinnings of the anorexigenic response by measuring transcript levels of relevant hypothalamic markers after acute treatment. After 2 days of treatment, RM-493 increased hypothalamic mRNA levels of *Pomc* (*P* < 0.01), neuropeptide Y (*Npy*) (*P* < 0.05) and agouti-related peptide (*Agrp*) (*P* < 0.05). The RM-493-induced increase in *Npy* and *Pomc* expression observed after 48-h acute treatment was normalized after 5 days of chronic treatment, whereas *Agrp* mRNA levels remained elevated (Fig3B). Strikingly, we observed higher expression of hypothalamic *Glp-1r* in mice treated with the combination therapy compared to liraglutide mono-therapy, after both 48-h acute treatment and 5 days of chronic treatment (Fig3A and B).

### Co-administration of RM-493 and liraglutide enhances the glycemic benefits compared to the respective mono-therapies

**Figure 4:**
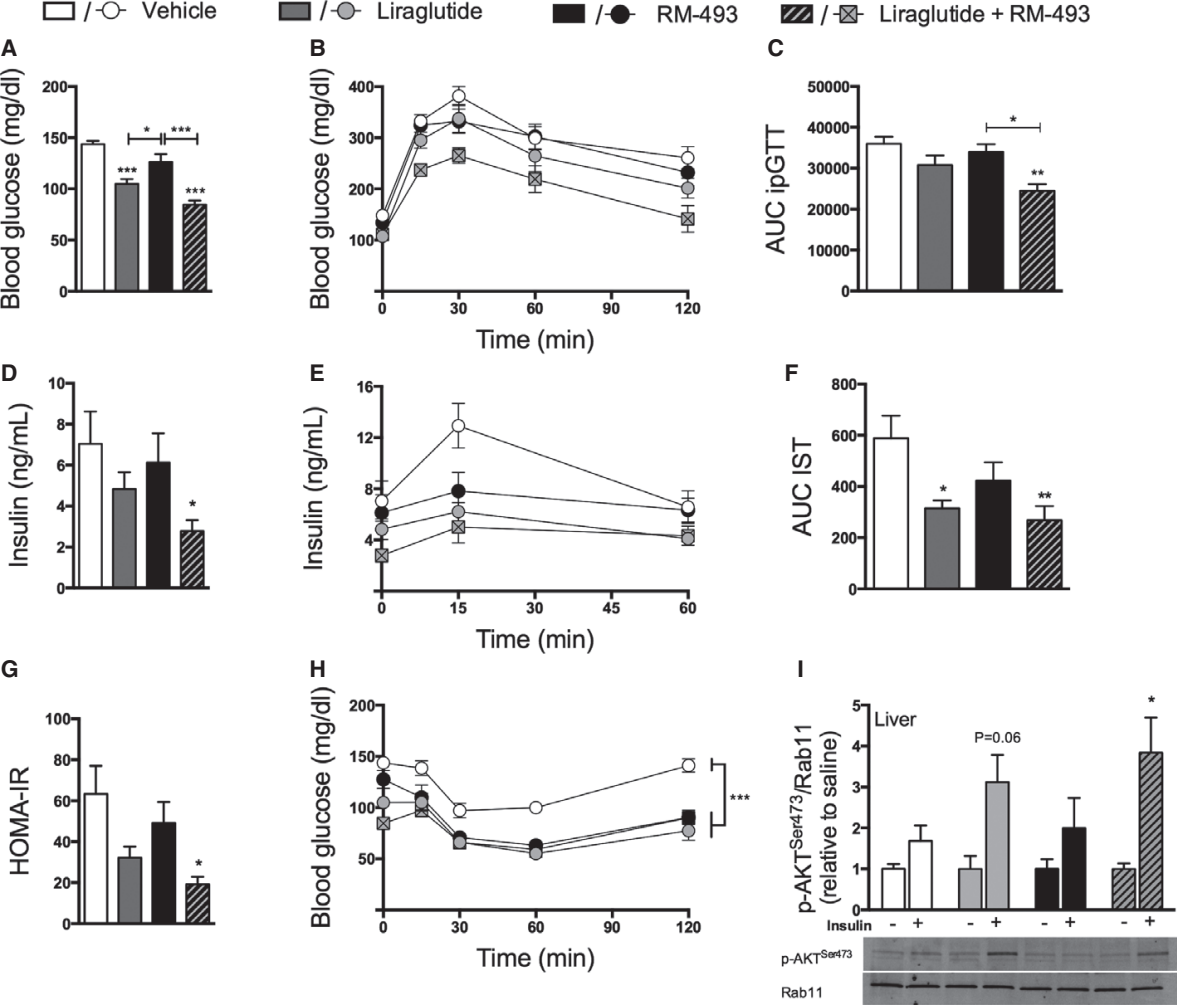
Effect of liraglutide and RM-493 co-treatment on glucose metabolism in DIO mice
A-I Glucose metabolic parameters were assessed following 5 days of treatment of DIO male mice with vehicle (white), liraglutide (10 nmol/kg) (gray), RM-493 (3.6 μmol/kg) (black), or liraglutide (10 nmol/kg) and RM-493 (3.6 μmol/kg) (checkered). (A) Fasted blood glucose levels, (B, C) glucose tolerance, (D) fasted insulin levels, (E, F) glucose-induced insulin secretion, (G) HOMA-IR, (H) insulin sensitivity and (I) hepatic phosphorylation of AKT (p-AKT^Ser473^) were analyzed. Compounds were administered by daily subcutaneous injections. To assess insulin-stimulated p-AKT^Ser473^, insulin (*n *=* *5) or saline (*n *=* *3) was injected 10 min prior to liver sampling. Data represent means ± SEM;*n *=* *8 in (A–H); *P < 0.05, **P < 0.01, ***P < 0.001.

After 5 days of treatment, fasting levels of blood glucose were equally reduced in mice treated with liraglutide and in mice treated with the combination therapy relative to vehicle controls (both *P* < 0.001; Fig4A). However, only the co-administration of liraglutide and RM-493, and not the corresponding individual treatments, improved glucose tolerance (*P* < 0.01) (Fig4B and C) and decreased fasting levels of insulin (*P* < 0.05) (Fig4D) at the doses tested here. Notably, glucose-stimulated insulin secretion was decreased following treatment with either mono-therapy and this effect was amplified by adjunctive administration of RM-493 and liraglutide, suggesting that the combination therapy enhances insulin sensitivity beyond the mono-therapies (Fig4E and F). Corroborating the enhanced insulin action in mice treated with the combination therapy, the homeostasis model assessment of insulin resistance (HOMA-IR) showed improved insulin sensitivity only in mice treated with the combination therapy (Fig4G). However, both individual treatments and their co-administration caused similar glucose clearance following a bolus insulin challenge (Fig4H). Nevertheless, insulin-induced phosphorylation of AKT^Ser473^ (p-AKT^Ser473^) was increased in liver samples of mice treated with the combination therapy but not in mice treated with either mono-therapy (Fig4I). Notably, no effect on p-AKT^Ser473^ induction between any group was observed in soleus muscle or epididymal white adipose tissue (Supplementary Fig S2A and B).

### Co-administration of RM-493 and liraglutide improves cholesterol metabolism compared to the respective mono-therapies

**Figure 5:**
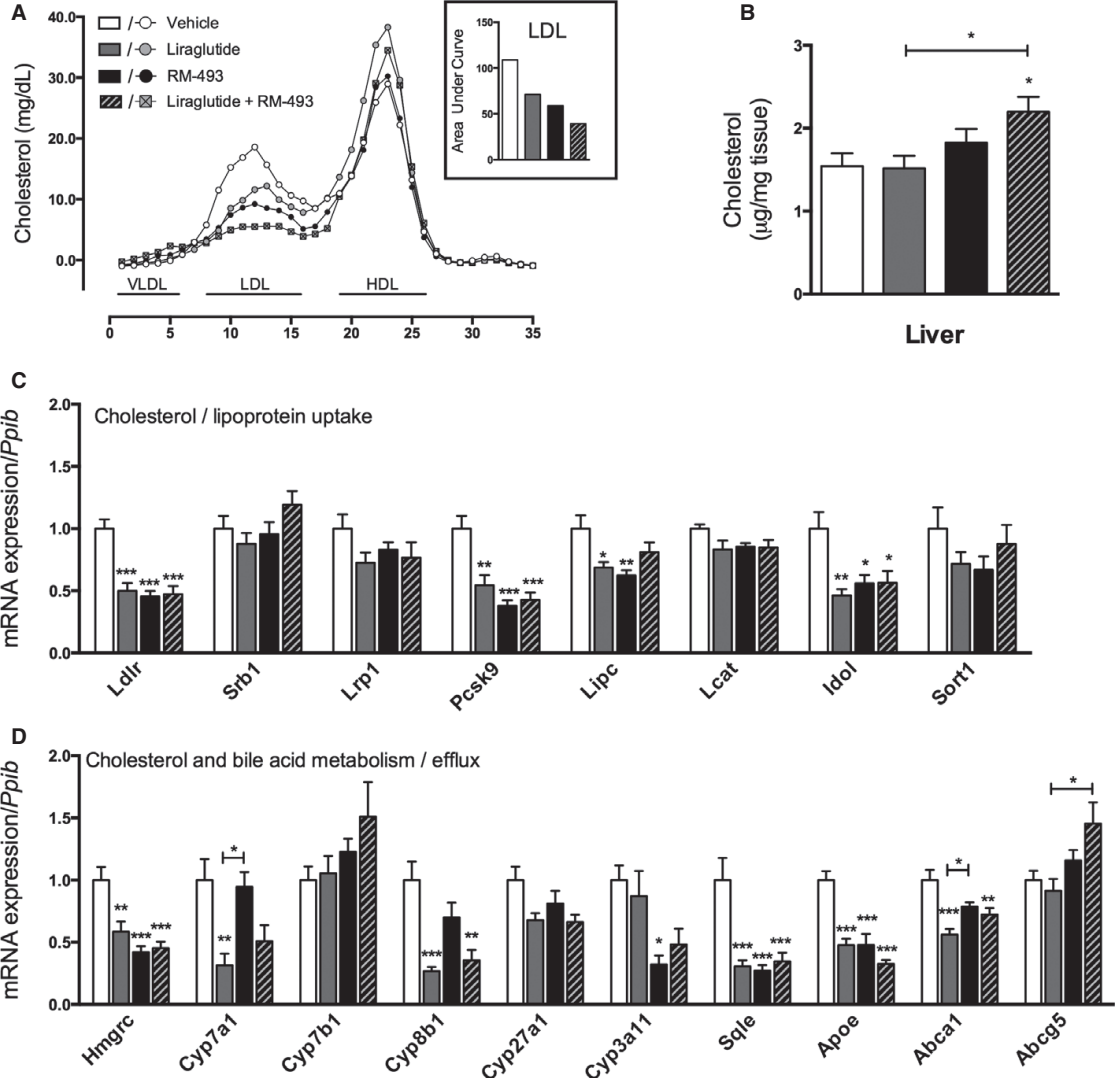
Effect of liraglutide and RM-493 co-treatment on cholesterol metabolism in DIO mice
A-D Cholesterol metabolic parameters were assessed following 5 days of treatment of DIO male mice with vehicle (white), liraglutide (10 nmol/kg) (gray), RM-493 (3.6 μmol/kg) (black), or liraglutide (10 nmol/kg) and RM-493 (3.6 μmol/kg) (checkered). (A) Plasma lipoprotein fractions, (B) liver cholesterol levels, (C) expression of genes implicated in hepatic cholesterol and lipoprotein uptake and (D) expression of genes implicated in cholesterol and bile acid metabolism and excretion were analyzed. Data represent means ± SEM;*n *=* *8; *P < 0.05, **P < 0.01, ***P < 0.001.

After 7 days of treatment, plasma levels of cholesterol were decreased in mice treated with liraglutide (*P* < 0.05), and this effect was enhanced by adjunctive administration of liraglutide and RM-493 (*P* < 0.01; Table1). The decreased levels of plasma cholesterol observed by the combination therapy were attributed to a substantial decrease (64% relative to vehicle) in low-density lipoprotein (LDL), and not high-density lipoprotein (HDL), which is enhanced compared to the effect of liraglutide (35%) and RM-493 (46%) alone (Fig5A). Whereas plasma levels of cholesterol were decreased, hepatic levels of cholesterol were increased following treatment with the combination therapy (Fig5B), suggesting that cholesterol uptake is enhanced. In line with this notion, the expression of *Pcsk9* and *Idol*, both negative regulators of the low-density lipoprotein receptor (*Ldlr*), were decreased in livers of mice treated with both mono-therapies and the combination therapy (Fig5C). Moreover, hepatic expression of *Hmgcr*, a key regulator of cholesterol biosynthesis, was decreased in the liver of mice treated with the mono-therapies and the combination therapy, whereas *Abcg5*, a regulator of secretion of cholesterol into bile, was increased by the combination therapy only (Fig5D). Conversely, the expression of several markers associated with cholesterol uptake and metabolism were decreased following treatment with both the mono-therapies and the combination therapy, indicating hepatic regulatory mechanisms controlling cholesterol uptake and turnover are activated.

**Table 1:** Effect of liraglutide and RM-493 co-treatment on plasma lipids in DIO mice.

Plasma lipids	Vehicle	Liraglutide	RM-493	Liraglutide and RM-493
Cholesterol (mg/dl)	220.0 ± 19.2	159.9 ± 9.8*	183.0 ± 9.3	141.2 ± 14.4**
Triglycerides (mg/dl)	79.5 ± 9.3	56.8 ± 5.1	89.8 ± 8.5	69.7 ± 3.1
NEFA (mg/dl)	26.1 ± 2.5	25.7 ± 1.9	27.0 ± 1.9	23.3 ± 0.9

Plasma lipid parameters in DIO mice maintained on a HFD and treated for 7 days with vehicle, liraglutide (10 nmol/kg), RM-493 (3.6 μmol/kg), or liraglutide (10 nmol/kg) and RM-493 (3.6 μmol/kg). Compounds were administered by daily subcutaneous injections. Data represent means ± SEM; *n *=* *8

* *P* < 0.05

** *P* < 0.01 versus vehicle.

### The energy and glucose metabolic benefits associated with the co-administration of RM-493 and liraglutide are preserved during prolonged treatment

**Figure 6:**
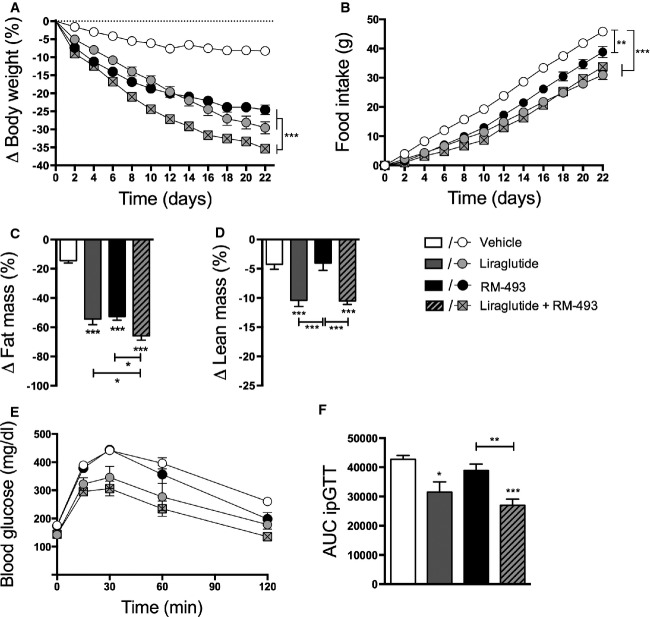
Effect of long-term liraglutide and RM-493 co-treatment on obesity and glucose metabolism in DIO mice
A-F 22 days of treatment of DIO male mice with vehicle (white), liraglutide (10 nmol/kg) (gray), RM-493 (3.6 μmol/kg) (black), or liraglutide (10 nmol/kg) and RM-493 (3.6 μmol/kg) (checkered). Effects on (A) body weight, (B) cumulative food intake, (C, D) body composition and (E, F) glucose tolerance. Compounds were administered by daily subcutaneous injections. Data represent means ± SEM;*n *=* *8; *P < 0.05, **P < 0.01, ***P < 0.001.

The beneficial effects of the RM-493 and liraglutide co-administration to improve body weight and glycemia were also confirmed in a more chronic setting. DIO mice co-treated for 22 days with liraglutide and RM-493 exhibited a 35.36 ± 0.82% weight loss, a significant amplification of the weight loss observed with either liraglutide (29.55 ± 1.66; *P* < 0.001) or RM-493 (24.55 ± 1.33%, *P* < 0.001) alone (Fig6A and Supplementary Fig S1D). In accordance with the 5-day study, we did not observe a difference in weight reduction between mice treated with either mono-therapy. The weight loss observed with both mono-therapies and the combination therapy was reflected in a reduction in food intake, albeit without significant differences between these treatment groups (Fig6B). Importantly, the prolonged treatment regime revealed a greater reduction of fat mass in mice treated with the combination therapy compared to mice treated with either agent alone (Fig6C). However, whereas RM-493 selectively reduced body fat mass, liraglutide and the co-administration of RM-493 and liraglutide caused a reduction in both lean and fat mass (Fig6D). Finally, in line with the 5-day study, the prolonged combinatorial treatment led not only to a greater loss in body weight but also to a greater improvement in glucose tolerance compared to either individual therapy (Fig6E and F).

## Discussion

Mimicking incretin biology has become a major therapeutic enterprise for treating type 2 diabetes (Garber, 2011). Emerging evidence supports that GLP-1R agonism may exhibit relevant weight-lowering properties in obese human subjects (Madsbad, 2014). However, the clinical applicability of GLP-1R analogs to reverse obesity and improve metabolic rearrangements is suboptimal and associated with dose-limiting adverse gastrointestinal events (Meier, 2012). In light of this, novel combination therapies that aim to broaden the therapeutic potential of GLP-1R agonism may provide unique opportunities for optimum treatment of metabolic disorders. In addition to complementary mechanisms of action, sub-threshold doses may sufficiently uphold pharmacological efficacy, while ameliorating adverse events associated with the respective mono-therapies. In the present study, we show that co-administration of the MC4R agonist RM-493, currently in phase II clinical trials, and the anti-diabetic GLP-1R agonist liraglutide yields superior metabolic outcomes relative to the corresponding individual therapies. The superior efficacy is due to a more pronounced effect to lower body fat mass, which is mediated by the combined anorectic actions of RM-493 and liraglutide along with the thermogenic properties arising from RM-493 action. In addition, adjunctive administration of RM-493 and liraglutide improves glucose tolerance and insulin sensitivity, lowers fasting levels of glucose and insulin and decreases cholesterol levels beyond what can be achieved with the corresponding mono-therapies.

In agreement with a recent study in non-human primates (Kievit *et al*, 2013), we confirm in mice that treatment with RM-493 as a stand-alone therapy can reverse diet-induced obesity. Importantly, we demonstrate for the first time that the metabolic effects of RM-493 on energy and glucose metabolism can be potentiated by adjunctive GLP-1R agonism. On average, DIO mice that were treated for 22 days with RM-493 obtained a 24.5% weight loss. This weight loss is a consequence of a reduction in body fat mass and not lean mass and is evident despite the maintenance of the mice on a high-fat, high-sucrose diet throughout the experiment, emphasizing the powerful weight-lowering properties of RM-493. Whereas the negative energy balance linked to RM-493 treatment appears to be a consequence of a parallel reduction in caloric intake and an increase in energy expenditure, liraglutide lowers body weight exclusively through reducing caloric intake. Co-administration of RM-493 and liraglutide complementarily decreases food intake without compromising the increased energy metabolism arising from MC4R activity and leads to a greater weight loss relative to each agonist as a mono-therapeutic. This enhanced weight loss is sustained under chronic treatment conditions.

At the cellular level, we observed an 83% reduction in hypothalamic *Glp1r* mRNA levels following acute (48 h) treatment with liraglutide. The liraglutide-mediated down-regulation of *Glp1r* was prevented when RM-493 was administered in combination with liraglutide. The putative role for RM-493 to maintain *Glp1r* expression was reinforced by a 5-day follow-up study, which demonstrated amplified hypothalamic *Glp1r* expression in mice treated with the combination therapy relative to vehicle- or liraglutide-treated mice. Accordingly, the superior metabolic efficacy of the combination therapy seems, at least in part, attributable to MC4R-mediated enhanced GLP-1R signaling. We also observed that RM-493 mono-therapy acutely increased both orexigenic (*Npy* and *Agrp*) and anorexigenic signals (*Pomc*) in the hypothalamus. The increase in *Agrp* and *Npy* coincides with a previous study showing that DIO mice treated with the MC4R ligand MTII have increased hypothalamic *Npy* and *Agrp* mRNA levels (Bluher *et al*, 2004). The amplified orexigenic signaling may represent a counter-regulatory physiological response imposed by the negative energy balance. Unlike Blüher *et al* 2004, however, who report that both short-term and long-term treatment with MT-II decreases hypothalamic *Pomc* mRNA levels, we find that RM-493 treatment transiently increased *Pomc* mRNA levels (78%). These conflicting *Pomc* expression data may relate to differences in sampling time, treatment regimes and/or treatment duration, but might also reflect differences in pharmacological properties between MT-II and RM-493. These central biomarkers of energy metabolism were normalized when liraglutide was administered in combination with RM-493 except for *Agrp*, which remained elevated. Whether the differential transcriptional regulations arise as a result of biological feedback at the circuitry level or as a consequence of direct cellular crosstalk between MC4R-positive neurons and GLP-1R-positive neurons remains to be established. Thus, future studies are warranted to define the molecular details of the combinatorial benefits of MC4R and GLP-1R agonism in energy metabolism.

In accordance with existing literature, we observed that treatment with liraglutide enhances glycemic control in DIO mice. In agreement with a previous report (Kievit *et al*, 2013), we identified that RM-493 improves diet-induced insulin resistance. Importantly, relative to RM-493 and liraglutide as stand-alone therapies, the glycemic benefits were amplified with their co-administration. Thus, to significantly reverse diet-induced glucose intolerance and hyperinsulinemia, co-treatment of RM-493 and liraglutide was required. The improved insulin sensitivity is primarily attributed to the liver as indicated by enhanced hepatic insulin signaling but not adipose- and skeletal muscle insulin signaling. Moreover, the combination of RM-493 and liraglutide enhanced the ability of both drugs to improve diet-induced disturbances in lipoprotein and cholesterol metabolisms, emphasizing the broad potential of combining liraglutide and RM-493 for treatment of the metabolic syndrome. The observation that the combination therapy has preferential efficacy to lower LDL but not HDL may hold translational value. These cumulative effects on hepatic metabolism suggest that a substantial contribution of the synergistic effects on glucose and lipid handling converges at the liver.

Taken together, our data reveal an unprecedented opportunity for treating diet-induced metabolic disorders with parallel pharmacological targeting of GLP-1R and MC4R. We show that combination therapy of the MC4R agonist RM-493, which has shown to be highly selective and safe in terms of its cardiovascular profile, with the GLP-1R agonist liraglutide potently reverses diet-induced obesity and improves glucose metabolism. Furthermore, the metabolic benefits associated with RM-493 and liraglutide combination therapy were superior to the metabolic benefits observed with the corresponding mono-therapies. These results align with a growing body of evidence showing that poly-pharmacological therapies may hold promise for the treatment of obesity and diabetes.

## Materials and Methods

### Animals and diet

Six- to eight-week-old male C57Bl/6 mice were given *ad libitum* access to a high-fat, high-sugar diet comprising 58% kcal fat (D12331; Research Diets, New Brunswick, NJ). The mice had free access to water and were maintained at 23 ± 1°C, constant humidity and on a 12-h light–dark cycle. Mice were maintained under these conditions for a minimum of 20 weeks before study initiation. At study start, mice were randomized into groups matched for body weight and body composition, with similar variance. No animals were excluded due to illness or outlier results; therefore, no exclusion determination was required. All animal studies were approved by the Animal Ethics Committee of the government of Upper Bavaria, Germany, and all experiments were performed according to the guidelines of the Institutional Animal Care and Use Committee of the Helmholtz Center Munich, Bavaria, Germany.

### *In vivo* pharmacological and energy metabolism studies

Compounds were administered subcutaneously 1 h before the onset of the dark phase. Co-administration of compounds was administered by single formulated injections. Researchers were not blinded to the treatment groups. Body composition (fat and lean mass) was analyzed using a magnetic resonance whole-body composition analyzer (EchoMRI, Houston, TX). Assessment of energy intake, energy expenditure and home-cage activity were performed using an indirect calorimetry system (TSE PhenoMaster, TSE Systems, Bad Homburg, Germany). Following 48 h of acclimatization, O_2_ consumption and CO_2_ production were measured every 10 min for a total of 115 h. Total energy expenditure was analyzed using ANCOVA, with body weight as covariate as previously suggested (Tschop *et al*, 2012). Home-cage locomotor activity was determined using an ActiMot infrared light beam system integrated in the calorimetry system.

### Glucose metabolism studies

Glucose tolerance was analyzed in 6-h-fasted mice, following an intraperitoneal challenge with 1.5 g glucose per kg body weight. Glucose levels were measured in blood sampled from the tail veins before (0 min) and at 15, 30, 60 and 120 min post injection. To measure glucose-stimulated insulin secretion, blood was collected from tail veins into EDTA-coated microvette tubes (Sarstedt) at time points 0, 15 and 60 min post glucose injection. Insulin sensitivity was assessed in 6-h-fasted mice following an intraperitoneal (IP) injection of 0.7 U of insulin (Actrapid) per kg body weight. Blood glucose levels were measured before (0 min) and at 15, 30, 60 and 120 min after the insulin injection.

### Chemical analysis of plasma and tissue

Blood samples were collected 2 h after the last injection. Blood was obtained from tail veins into EDTA-coated microvette tubes (Sarstedt), centrifuged at 5,000 *g*, and plasma was stored at −80°C. Commercially available kits were used to measure plasma levels of cholesterol (Wako Chemicals, Neuss, Germany), free fatty acids, NEFA (Wako Chemicals, Neuss, Germany), TG (Wako Chemicals, Neuss, Germany) and insulin (ALPCO Diagnostics, Salem, NH) as per the manufacturer's instructions. For lipoprotein separation, samples from eight animals per group were pooled (0.20 ml) and subjected to fast-performance liquid chromatography (FPLC) gel filtration on two Superose 6 columns connected in series as described previously (Hofmann *et al*, 2008). Hepatic lipids were extracted as described previously (Folch *et al*, 1957), and cholesterol content was quantified using a commercially available kit (Thermo Scientific, Middletown, VA).

### Gene expression analysis

Gene expression profiling in the hypothalamus was performed in mice treated with compounds for 2 or 5 consecutive days, whereas gene expression profiling in the liver was performed in mice treated with compounds for 5 consecutive days. For tissue collection, mice were fasted for 4 h and treated with compounds 2 h prior to tissue collection. Hypothalamic and liver gene expression was profiled with quantitative real-time RT–PCR using either TaqMan single probes or SYBR Green with validated primers. The relative expression of the selected hypothalamic genes was normalized to the reference gene hypoxanthine-guanine phosphoribosyltransferase (*Hprt*) and, in the selected hepatic genes, to the reference gene peptidyl-prolyl cis-trans isomerase b (*Ppib*).

### Western blotting

For protein quantification, mice were euthanized by cervical dislocation 10 min after an IP injection of 1 mg of insulin (Apidra) per kg body weight or saline, and tissues (liver, soleus muscle and epididymal white adipose tissue) were rapidly frozen in liquid nitrogen and stored at −80°C until further analysis. Tissues were lysed in ice-cold RIPA lysis buffer (Sigma-Aldrich, Germany) containing protease (Thermo Scientific, Rockford, IL) inhibitor using Qiagen tissue homogenizer.

Proteins were separated on Criterion gel (Bio-Rad, Germany) and transferred on to nitrocellulose membranes. Membranes were incubated with primary antibody (Pathscan Multiplex, Cell Signaling, Danvers, MA) overnight (dilution: 1:1,000), and fluorescent-labeled secondary antibody (IRDye® 800CW goat anti-rabbit IgG, LI-COR Biosciences, Nebraska, USA) was used to visualize phosphorylated proteins using LI-COR imaging system (dilution 1:15,000).

### Statistics

Statistical differences were performed on data distributed in a normal pattern using one- or two-way ANOVA followed by Bonferroni's *post hoc* analysis as appropriate, or an unpaired two-tailed Student's *t*-test. All results are presented as mean ± SEM, and *P *<* *0.05 was considered significant. Group size estimations were determined by power calculation to minimally yield an 80% chance to detect a significant change in body weight of *P *<* *0.05. Exact *P*-values are provided in Supplementary Table S1.

## Supporting Information

Supplementary Information

Review Process File
