# Supplementary material for: Dual melanocortin-4 receptor and GLP-1 receptor agonism amplifies metabolic benefits in diet-induced obese mice
Source: EMBO Mol Med. 2015 Feb 4;7(3):288–98. doi: 10.15252/emmm.201404508 (PMC4364946; doi:10.15252/emmm.201404508)
Supplement: Supplementary file 1 [file emmm0007-0288-sd1.pdf]

Supplementary information

Dual melanocortin-4 receptor and GLP-1 receptor agonism amplifies metabolic benefits in diet-induced obese mice

Christoffer Clemmensen, Brian Finan, Katrin Fischer, Robby Zachariah Tom, Beata Legutko, Laura Sehrer, Daniela Heine, Niklas Grassl, Carola W. Meyer, Bart Henderson, Susanna M. Hofmann, Matthias H. Tschöp, Lex H.T. Van der Ploeg and Timo D. Müller

Contents:

1) Supplementary figure legends

2) Figure S1
Effect on liraglutide and RM-493 co-treatment on body weight and body composition in DIO mice.

3) Figure S2
Effect on liraglutide and RM-493 co-treatment on insulin-induced phosphorylation of AKT in DIO mice.

4) Supplementary Table S1
P-values of statistical significant findings

Supplementary figure legends

Figure S1. Effect of liraglutide and RM-493 co-treatment on body weight and body composition in DIO mice. (A-C) Five-day treatment and (D-F) 22-day treatment of DIO male mice with vehicle (white), liraglutide (10 nmoles/kg) (grey), RM-493 (3.6 µmoles/kg) (black) or liraglutide (10 nmoles/kg) and RM-493 (3.6 µmoles/kg) (checkered). Effects on (A) body weight and (B and C) body composition following 5 days of treatment. Effects on (D) body weight and (E and F) body composition following 22 days of treatment. Compounds were administered by daily subcutaneous injections. Data represent means ± SEM; *n* = 8; *P < 0.05, **P < 0,01, ***P < 0.001.

Figure S2. Effect of liraglutide and RM-493 co-treatment on insulin-induced phosphorylation of AKT in DIO mice. Phosphorylation of AKT (p-AKT^{Ser473}) was assessed in (A) epididymal white adipose tissue (eWAT) and (B) soleus muscle in DIO mice treated for 5 days with vehicle (white), liraglutide (10 nmoles/kg) (grey), RM-493 (3.6 µmoles/kg) (black) or liraglutide (10 nmoles/kg) and RM-493 (3.6 µmoles/kg) (checkered). Compounds were administered by daily subcutaneous

injections and the last injection was provided 2h prior to tissues sampling. Insulin (n = 5) or saline (n = 3) was injected 10 min prior to tissue removal. Data represent means ± SEM.

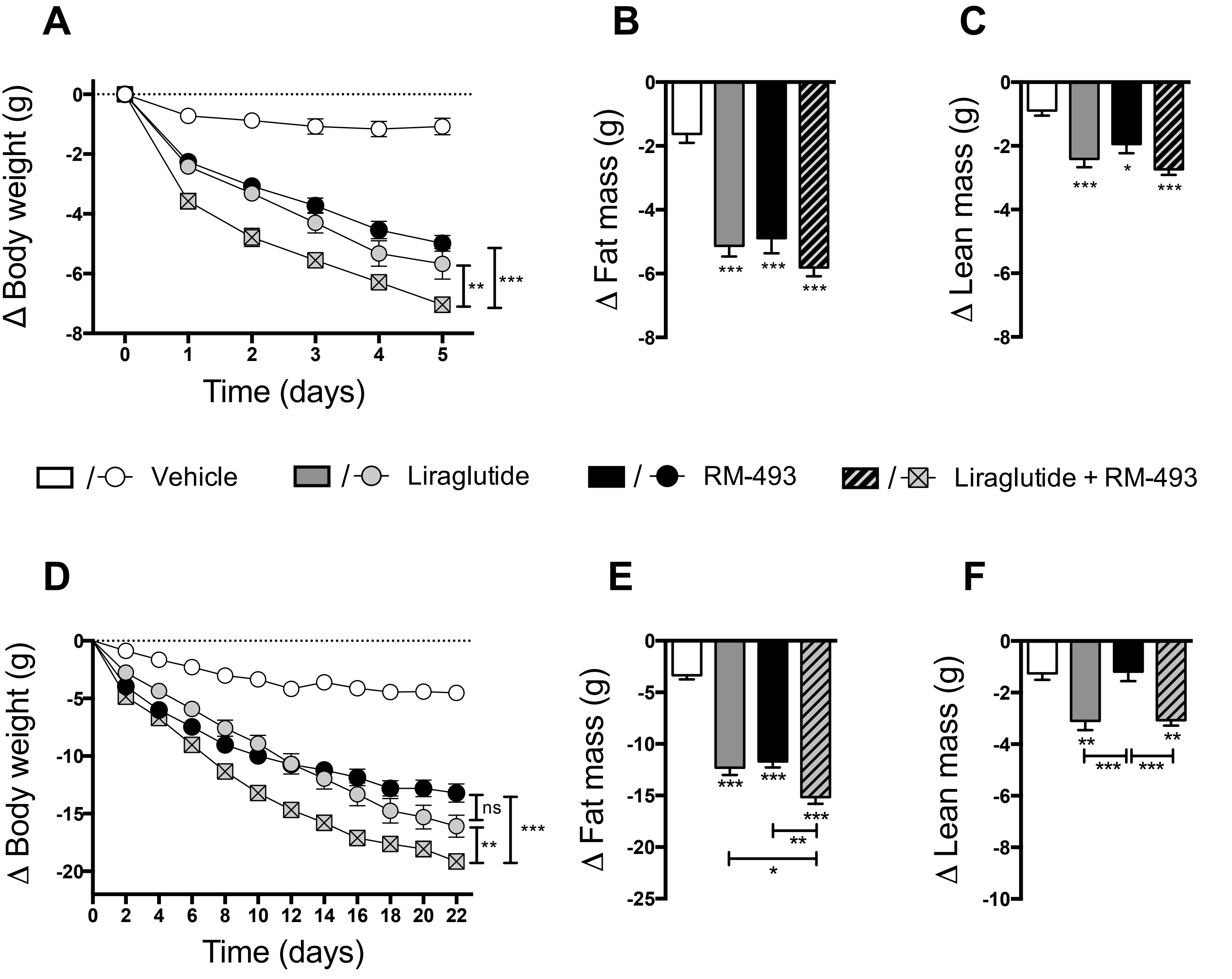

A
B
C
Δ Body weight (g)
Time (days)
Δ Fat mass (g)
Δ Lean mass (g)
Vehicle
Liraglutide
RM-493
Liraglutide + RM-493
D
E
F
ns

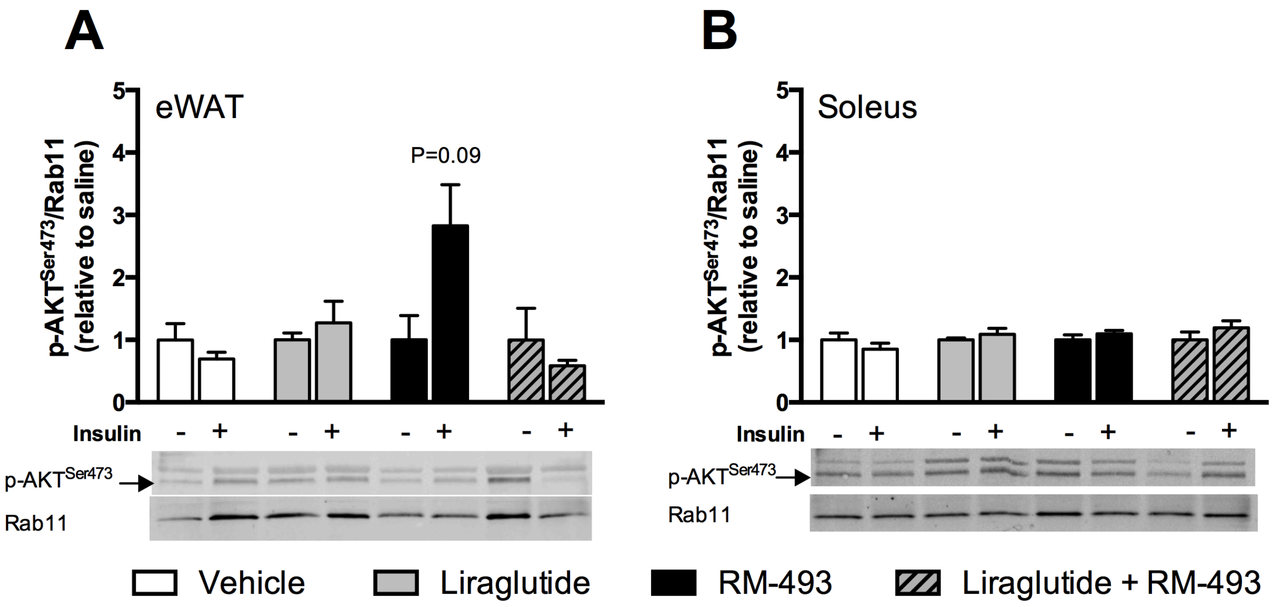
A
eWAT
p-AKT^Ser473/Rab11 (relative to saline)
P=0.09
Insulin - + - + - + - +
p-AKT^Ser473
Rab11
B
Soleus
p-AKT^Ser473/Rab11 (relative to saline)
Insulin - + - + - + - +
p-AKT^Ser473
Rab11
Vehicle
Liraglutide
RM-493
Liraglutide + RM-493

Supplementary Table 1.

P-values of statistical significant findings

Vhcl; Vehicle
Lira; Liraglutide
RM; RM-493
Combo; Liraglutide + RM-493

Figure		Comparison	P-value		
1	A	Vhcl vs. Lira	***	0.0001	2way ANOVO -Bonferroni
		Vhcl vs. RM	***	0.0001	2way ANOVO -Bonferroni
		Lira vs. Combo	***	0.0006	2way ANOVO -Bonferroni
		RM vs. Combo	***	0.0001	1way ANOVA -Bonferroni
	B	Vhcl vs. Lira	***	0.0001	1way ANOVA -Bonferroni
		Vhcl vs. RM	***	0.0001	1way ANOVA -Bonferroni
		Vhcl vs. Combo	***	0.0001	1way ANOVA -Bonferroni
	C	Vhcl vs. Lira	***	0.0001	1way ANOVA -Bonferroni
		Vhcl vs. RM	**	0.0068	1way ANOVA -Bonferroni
		Vhcl vs. Combo	***	0.0001	1way ANOVA -Bonferroni
		RM vs. Combo	*	0.0325	1way ANOVA -Bonferroni
2	B	Vhcl vs. RM	***	0.0001	1way ANOVA -Bonferroni
		Vhcl vs. Combo	***	0.0001	1way ANOVA -Bonferroni
		Lira vs. RM	***	0.0001	1way ANOVA -Bonferroni
		Lira vs. Combo	***	0.0001	1way ANOVA -Bonferroni
	C	Total Vhcl vs. Lira	***	0.0001	1way ANOVA -Bonferroni
		Total Vhcl vs. RM	***	0.0001	1way ANOVA -Bonferroni
		Total Vhcl vs. Combo	***	0.0001	1way ANOVA -Bonferroni
		Dark Vhcl vs. Lira	***	0.0001	1way ANOVA -Bonferroni
		Dark Vhcl vs. RM	***	0.0001	1way ANOVA -Bonferroni
		Dark Vhcl vs. Combo	***	0.0001	1way ANOVA -Bonferroni
		Dark RM vs. Combo	*	0.0451	1way ANOVA -Bonferroni
		Light Vhcl vs. Lira	***	0.0001	1way ANOVA -Bonferroni
		Light Vhcl vs. RM	***	0.0009	1way ANOVA -Bonferroni
		Light Vhcl vs. Combo	***	0.0001	1way ANOVA -Bonferroni
	E	RM vs. Combo	**	0.0096	2way ANOVO -Bonferroni
		Lira vs. Combo	*	0.0198	2way ANOVO -Bonferroni
	F	Total Vhcl vs. Lira	***	0.0001	1way ANOVA -Bonferroni
		Total Vhcl vs. RM	***	0.0001	1way ANOVA -Bonferroni
		Total Vhcl vs. Combo	***	0.0001	1way ANOVA -Bonferroni
		Dark Vhcl vs. Lira	***	0.0001	1way ANOVA -Bonferroni
		Dark Vhcl vs. RM	***	0.0001	1way ANOVA -Bonferroni
		Dark Vhcl vs. Combo	***	0.0001	1way ANOVA -Bonferroni
		Dark RM vs. Combo	*	0.0389	1way ANOVA -Bonferroni
		Light Vhcl vs. Lira	**	0.0023	1way ANOVA -Bonferroni
		Light Vhcl vs. Combo	**	0.0032	1way ANOVA -Bonferroni
	G	Dark Vhcl vs. Lira	*	0.0204	1way ANOVA -Bonferroni
3	A	GLP1R Vhcl vs. Lira	**	0.0030	1way ANOVA -Bonferroni
		GLP1R Vhcl vs. Combo	#	0.0157	1way ANOVA -Bonferroni
		POMC Vhcl vs. RM	**	0.0057	1way ANOVA -Bonferroni
		POMC Lira vs. RM	##	0.0004	1way ANOVA -Bonferroni
		NPY Vhcl vs. RM	*	0.0166	1way ANOVA -Bonferroni
		NPY Lira vs. RM	###	0.0002	1way ANOVA -Bonferroni
		NPY RM vs. Combo	+	0.0479	1way ANOVA -Bonferroni
		AGRP Vhcl vs. RM	*	0.0151	1way ANOVA -Bonferroni
		AGRP Lira vs. RM	##	0.0023	1way ANOVA -Bonferroni
		AGRP Lira vs. Combo	#	0.0108	1way ANOVA -Bonferroni
	B	GLP1R Vhcl vs. Combo	**	0.0072	1way ANOVA -Bonferroni
		GLP1R Lira vs. Combo	##	0.0038	1way ANOVA -Bonferroni
		MC4R Vhcl vs. RM	§	0.0006	1way ANOVA -Bonferroni
		MC4R Vhcl vs. Combo	§	0.007	1way ANOVA -Bonferroni
		AGRP Vhcl vs. RM	*	0.0221	1way ANOVA -Bonferroni
		AGRP Vhcl vs. Combo	**	0.0013	1way ANOVA -Bonferroni
		AGRP Lira vs. Combo	#	0.0471	1way ANOVA -Bonferroni
4	A	Vhcl vs. Lira	***	0.0001	1way ANOVA -Bonferroni
		Vhcl vs. Combo	***	0.0001	1way ANOVA -Bonferroni
		Lira vs. RM	*	0.0426	1way ANOVA -Bonferroni
		RM vs. Combo	***	0.0001	1way ANOVA -Bonferroni
	C	Vhcl vs. Combo	**	0.0017	1way ANOVA -Bonferroni
		RM vs. Combo	*	0.0116	1way ANOVA -Bonferroni
	D	Vhcl vs. Combo	*	0.0458	1way ANOVA -Bonferroni
	F	Vhcl vs. Lira	*	0.0347	1way ANOVA -Bonferroni
		Vhcl vs. Combo	**	0.0097	1way ANOVA -Bonferroni
	G	Vhcl vs. Combo	*	0.0104	1way ANOVA -Bonferroni
	H	Vhcl vs. Lira	***	0.0001	2way ANOVO -Bonferroni

		Vhcl vs. RM	***	0.0001	2way ANOVO -Bonferroni
		Vhcl vs. Combo	***	0.0001	2way ANOVO -Bonferroni
	I	Combo saline vs. Insulin	*	0.0478	Student's t-test
5	B	Vhcl vs. Combo	*	0.0256	1way ANOVA -Bonferroni
		Lira vs. Combo	*	0.0252	1way ANOVA -Bonferroni
	C	LDLR Vhcl vs. Lira	***	0.0001	1way ANOVA -Bonferroni
		LDLR Vhcl vs. RM	***	0.0001	1way ANOVA -Bonferroni
		LDLR Vhcl vs. Combo	***	0.0001	1way ANOVA -Bonferroni
		PCSK9 Vhcl vs. Lira	**	0.0011	1way ANOVA -Bonferroni
		PCSK9 Vhcl vs. RM	***	0.0001	1way ANOVA -Bonferroni
		PCSK9 Vhcl vs. Combo	***	0.0001	1way ANOVA -Bonferroni
		LIPC Vhcl vs. Lira	*	0.0302	1way ANOVA -Bonferroni
		LIPC Vhcl vs. RM	**	0.0063	1way ANOVA -Bonferroni
		IDOL Vhcl vs. Lira	**	0.0017	1way ANOVA -Bonferroni
		IDOL Vhcl vs. RM	*	0.0124	1way ANOVA -Bonferroni
		IDOL Vhcl vs. Combo	*	0.0137	1way ANOVA -Bonferroni
	D	HMGRC Vhcl vs. Lira	**	0.0032	1way ANOVA -Bonferroni
		HMGRC Vhcl vs. RM	***	0.0001	1way ANOVA -Bonferroni
		HMGRC Vhcl vs. Combo	***	0.0001	1way ANOVA -Bonferroni
		CYP7A1 Vhcl vs. Lira	**	0.0058	1way ANOVA -Bonferroni
		CYP7A1 Lira vs. RM	*	0.0126	1way ANOVA -Bonferroni
		CYP8B1 Vhcl vs. Lira	***	0.0004	1way ANOVA -Bonferroni
		CYP8B1 Vhcl vs. Combo	**	0.0013	1way ANOVA -Bonferroni
		CYP3A11 Vhcl vs. RM	*	0.0147	1way ANOVA -Bonferroni
		SQLE Vhcl vs. Lira	***	0.0001	1way ANOVA -Bonferroni
		SQLE Vhcl vs. RM	***	0.0001	1way ANOVA -Bonferroni
		SQLE Vhcl vs. Combo	***	0.0001	1way ANOVA -Bonferroni
		APOE Vhcl vs. Lira	***	0.0001	1way ANOVA -Bonferroni
		APOE Vhcl vs. RM	***	0.0001	1way ANOVA -Bonferroni
		APOE Vhcl vs. Combo	***	0.0001	1way ANOVA -Bonferroni
		ABCA1 Vhcl vs. Lira	***	0.0001	1way ANOVA -Bonferroni
		ABCA1 Vhcl vs. Combo	**	0.0095	1way ANOVA -Bonferroni
		ABCA1 Lira vs. RM	*	0.0482	1way ANOVA -Bonferroni
		ABCG5 Lira vs. Combo	*	0.0131	1way ANOVA -Bonferroni
6	A	RM vs. Combo	***	0.0002	2way ANOVO -Bonferroni
		Lira vs. Combo	***	0.0003	2way ANOVO -Bonferroni
	B	Vhcl vs. Lira	***	0.0001	2way ANOVO -Bonferroni
		Vhcl vs. RM	**	0.0013	2way ANOVO -Bonferroni
		Vhcl vs. Combo	***	0.0001	2way ANOVO -Bonferroni
	C	Vhcl vs. Lira	***	0.0001	1way ANOVA -Bonferroni
		Vhcl vs. RM	***	0.0001	1way ANOVA -Bonferroni
		Vhcl vs. Combo	***	0.0001	1way ANOVA -Bonferroni
		Lira vs. Combo	*	0.0469	1way ANOVA -Bonferroni
		RM vs. Combo	*	0.0156	1way ANOVA -Bonferroni
	D	Vhcl vs. Lira	***	0.0006	1way ANOVA -Bonferroni
		Vhcl vs. Combo	***	0.0005	1way ANOVA -Bonferroni
		Lira vs. RM	***	0.0004	1way ANOVA -Bonferroni
		RM vs. Combo	***	0.0003	1way ANOVA -Bonferroni
	F	Vhcl vs. Lira	*	0.0156	1way ANOVA -Bonferroni
		Vhcl vs. Combo	***	0.0005	1way ANOVA -Bonferroni
		RM vs. Combo	**	0.0093	1way ANOVA -Bonferroni

Table	1	Cholesterol Vhcl vs. Lira	*	0.0211	1way ANOVA -Bonferroni
		Cholesterol Vhcl vs. Combo	**	0.0017	1way ANOVA -Bonferroni

S1	A	Lira vs. Combo	**	0.0065	2way ANOVO -Bonferroni
		RM vs. Combo	***	0.0001	2way ANOVO -Bonferroni
	B	Vhcl vs. Lira	***	0.0001	1way ANOVA -Bonferroni
		Vhcl vs. RM	***	0.0001	1way ANOVA -Bonferroni
		Vhcl vs. Combo	***	0.0001	1way ANOVA -Bonferroni
	C	Vhcl vs. Lira	***	0.0003	1way ANOVA -Bonferroni
		Vhcl vs. RM	*	0.0151	1way ANOVA -Bonferroni
		Vhcl vs. Combo	***	0.0001	1way ANOVA -Bonferroni
	D	Lira vs. Combo	**	0.0014	2way ANOVO -Bonferroni
		RM vs. Combo	***	0.0006	2way ANOVO -Bonferroni
	E	Vhcl vs. Lira	***	0.0001	1way ANOVA -Bonferroni
		Vhcl vs. RM	***	0.0001	1way ANOVA -Bonferroni
		Vhcl vs. Combo	***	0.0001	1way ANOVA -Bonferroni
		Lira vs. Combo	*	0.0117	1way ANOVA -Bonferroni
		RM vs. Combo	**	0.0018	1way ANOVA -Bonferroni
	F	Vhcl vs. Lira	**	0.0011	1way ANOVA -Bonferroni
		Vhcl vs. Combo	**	0.0013	1way ANOVA -Bonferroni
		Lira vs. RM	***	0.0007	1way ANOVA -Bonferroni
		RM vs. Combo	***	0.0009	1way ANOVA -Bonferroni
