# Supplementary material for: Dual melanocortin-4 receptor and GLP-1 receptor agonism amplifies metabolic benefits in diet-induced obese mice
Source: EMBO Mol Med. 2015 Feb 4;7(3):288–98. doi: 10.15252/emmm.201404508 (PMC4364946; doi:10.15252/emmm.201404508)
Supplement: Supplementary file 2 [file emmm0007-0288-sd2.pdf]

EMBO Molecular Medicine

Dual melanocortin-4 receptor and GLP-1 receptor agonism amplifies metabolic benefits in diet-induced obese mice

Christoffer Clemmensen, Brian Finan, Katrin Fischer, Beata Legutko, Laura Sehrer, Daniela Heine, Niklas Grassl, Carola Meyer, Bart Henderson, Matthias Tschöp, Lex Van der Ploeg

Corresponding author: Timo Müller, Helmholtz Zentrum Munich, IDO

Review timeline:

	Submission date:	08 August 2014
	Accepted:	13 January 2015

Editor: Céline Carret

Transaction Report:

No Peer Review Process File is available with this article, as the authors have chosen not to make the review process public in this case.
